# Psychometric properties of the Malay version of the diabetes empowerment scale among hospital Serdang type 2 diabetes mellitus patients using exploratory factor analysis

**DOI:** 10.1186/s12955-020-1280-0

**Published:** 2020-02-07

**Authors:** Siew Mooi Ching, Anne Yee, Ping Yein Lee, Vasudevan Ramachandran, Khai Mun Shum, Nur Fati’Izzati Ismael, Wan Aliaa Wan Sulaiman, Fan Kee Hoo, Yoke Loong Foo, Kai Wei Lee, Mahmoud Danaee

**Affiliations:** 1 Department of Family Medicine, Faculty of Medicine and Health Sciences, Universiti Putra Malaysia, Serdang, Malaysia; 2 Malaysian Research Institute on Ageing, Universiti Putra Malaysia, 43400 Serdang, Selangor Darul Ehsan Malaysia; 3 Department of Psychological Medicine, Faculty of Medicine, University of Malaya, 50603 Kuala Lumpur, Malaysia; 4 Faculty of Medicine and Health Sciences, Universiti Putra Malaysia, Serdang, Selangor Malaysia; 5 Department of Medicine, Faculty of Medicine and Health Sciences, Universiti Putra Malaysia, Serdang, Selangor Malaysia; 6 Department of Social and Preventive Medicine, Faculty of Medicine, University of Malaya, 50603 Kuala Lumpur, Malaysia

**Keywords:** Diabetes education, Empowerment, Type 2 diabetes, Malaysia

## Abstract

**Background:**

This study was initiated to examine the psychometric components of the Diabetes Empowerment Scale (DES) by translating and validating the scale into the Malay language (DES-M) which is the main language spoken in Malaysia. This study can determine the level of empowerment among diabetic patients towards diabetes management. In addition, the reliability and validity of the DES-M was also demonstrated.

**Methods:**

A total of 151 patients with type 2 diabetes mellitus were recruited (between June 2016 and October 2016) to complete sets of questionnaires, which were DES-M, the Malay versions of the Diabetes Quality of Life (DQOL) for Adults and Summary of Diabetes Self Care Activities Questionnaire (SDSCA). Confirmatory and Exploratory factor analysis (CFA and EFA) were carried out to determine the factor structures of the DES-M.

**Results:**

There were 100 males and 51 females with ages ranging from 19 to 81 years old (55 ± 13) included in this study. The instrument displayed good internal consistency (Cronbach’s α =0.920) and the respective coefficients ranged from 0.65–0.84. Discriminant validity showed adequate correlations ranged from 0.257–0.744. Concurrent validity with SDSCA (Pearson’s correlation = 0.313, *p* = 0.012). Predictive validity with DQOL (B = 0.27, *p* = 0.016). CFA indicated that four factor model of the DES-M has good fit to the data.

**Conclusion:**

This study indicates that the DES-M has a good internal consistency and validity. Therefore, it is a valid and reliable instrument for assessing empowerment score among patients with diabetes in Malaysia.

**Trial registration:**

NMRR-16-805-30503 (IIR).

## Background

Diabetes mellitus (DM) is an important public health problem in view of there is a tremendous increment in diabetes prevalence for the past two decades. In 20 years (1986 to 2006), the prevalence of DM has doubled from 6 to 12% [1]. Diabetes also has a high mortality and morbidity [2]. However the control rate among patients with DM in Malaysia is still inadequate [3].

Diabetes empowerment is a process of self-care of their diabetic condition. Patient with good empowerment has shown to have better health outcome [4, 5]. Thus, diabetes empowerment is considered a core component of diabetes care [6, 7]. Diabetes empowerment is integrated into healthcare system, which it involves educational intervention to increase one’s ability to think cautiously in the process of gain control over their diabetic condition subsequently improve their health-related outcomes [8]. Empowerment increases patients capacity to help themselves in their diabetes care [9], particularly to improve compliance rate of patients in practicing healthy lifestyle and medication uptake [10, 11]. Studies have shown patients who are involved in decisions regarding their care and management have better outcomes compared to those who are not [12, 13]. Furthermore, those patients who succeed in self-empowered will motivate other patients with diabetes in achieving a better glucose control [14, 15].

Diabetes Empowerment Scale (DES) has been developed as a tool to assess the self-empowerment [16]. Other than DES, there are another two instruments which are Diabetes Self-Management Questionnaire (DSMQ) [17] and Summary of diabetes self-care activities measure (SDSCA) [18, 19]. DSMQ is a 16-items questionnaire developed by Schmitt et al., 2013, to assess behaviors associated with metabolic control within common treatment regimens for type 1 and type 2 diabetes in adult patients. Validation of DSMQ showed it is reliable instrument with Cronbach’s alpha value of 0.84 (0.77 for subscale glucose management; 0.77 for dietary control; 0.76 for physical activity; and 0.57 for health care use). This questionnaire was performed among in-patient at a tertiary referral centres for diabetes, which the patients were having problems of diabetes treatment and poor glycaemic control with relatively long average diabetes duration and high prevalence of late complications. It is therefore the validation results couldn’t be generalized among the general diabetic population [17]. SDSCA is an 11-items questionnaire developed by Toobert et al., 1994 [18], later revised by Toobert et al., 2000 [19], assessing the following aspects of the diabetes regimen: general diet, specific diet, exercise, blood-glucose testing, foot care, and smoking. The average inter-item correlations within scales were high (mean = 0.47), with the exception of specific diet; test-retest correlations were moderate (mean = 0.40). Correlations with other measures of diet and exercise generally supported the validity of the SDSCA subscales (mean = 0.23) [18, 19]. Many translations of the SDSCA have been validated such as Spanish [4], German [20], Arabic [21], Turkish [22], Korean [23] including Malay [24]. The validated SDSCA in malay version by Bujang et al. (2016), reported that the cronbach’s alpha for the main domains based on the fieldwork were between 0.651 and 0.905 [24].

We chose DES for our study as the DES-28 questionnaire has the highest Cronbach’s alpha value compared to other scales (Cronbach’s alpha = 0.96) which is a good internal consistency [16]. The Cronbach’s alpha of each subscale was 0.93 for “managing the psycho-social aspects of diabetes”; 0.81 for “assessing dissatisfaction and readiness to change”; and 0.91 for “setting and achieving diabetes goals” [16]. On top of that, DES scale is designed specifically to measure the empowerment of the patient with diabetes. To the best of researcher knowledge, a validated empowerment questionnaire in local setting is unprecedented. This study aimed to translate the DES into the Malay language and to study the psychometric properties of the Malay version of the DES-M to facilitate its use for further research in the local setting.

## Methods

### Study design and setting

This was a cross-sectional study, in which the data was collected from self-administered questionnaire that was distributed to patients with Type 2 diabetes mellitus in Hospital Serdang.

### Procedure

#### Stage 1

The authors obtained the permission to use the English version of the DES-28 from Michigan Diabetes Research Center [16]. Translation from English to Malay was performed by a bilingual language expert and a back translation was performed by another bilingual language expert who is not to aware of the concept of the questionnaire. The process of translation and adaptation of instrument of this study followed World Health Organization guidelines [25]. Discrepancies between the original and the back translation was discussed, and adjustments are made accordingly. A final version of translated DES, which we called as draft of DES-M was generated by an expert panel composed of one psychologist and three senior family physicians, all of whom were qualified professionals regarding use of psychometric instruments and all of whom had clinical experience with management of diabetes.

#### Stage 2

The final version of DES-M was distributed among 201 Type 2 diabetes mellitus patients in Hospital Serdang after receiving a full explanation of the nature and confidentiality of the study and a written consent. The first draft of DES-M was tested by pilot study among 22 type 2 diabetic patients in Hospital Serdang to identify any flaws in the questionnaire. Any words that the patients did not understand or considered inappropriate and remarks from the patients was noted and corrected. The finalized version of DES-M was further reviewed by the before-mentioned specialists.

#### Stage 3

The final version of DES-M was distributed among 151 type 2 diabetic patients in Hospital Serdang after receiving a full explanation of the nature and confidentiality of the study and a written informed consent. Patients personal and socio-demographic data, other comorbidities (hypertension asthma, dyslipidemia, ischaemic heart disease), smoking status, and diabetes education experience were taken. The non-response rate was 25%. Other than the DES-M (Table 1), the Diabetes Quality of Life Questionnaire (DQOL) for Adults and Summary of Diabetes Self Care Activities Questionnaire (SDSCA) was distributed to the respondents to test the predictive validity and convergent validity, respectively.

**Table 1:** Socio-demographic and characteristics of study participants (*N* = 151)

Characteristics	N (%)
Gender	
Male	100 (66.2)
Female	51 (33.8)
Ethnicity	
Malays	93 (61.6)
Chinese	18 (11.9)
Indians	37 (24.5)
Others	3 (2.0)
Education Status	
Secondary education and below	97 (64.2)
Tertiary education	54 (35.8)
Marital status	
Single	13 (8.6)
Married	138 (91.4)
Hypertension	
Yes	116 (76.8)
No	35 (23.2)
Asthma	
Yes	16 (10.6)
No	135 (89.4)
Dyslipidaemia	
Yes	91 (60.3)
No	60 (39.7)
Ischaemic geart disease	
Yes	50 (33.1)
No	101 (66.9)
Smoking status	
Yes	19 (12.6)
No	132 (87.4)
Diabetes education	
Yes	51 (33.8)
No	100 (66.2)

### Instruments

The DES is a 28-item self-reported scale used to measure the psychosocial self-efficacy of diabetic patients. It consists of subscales reflecting three domains; managing psychosocial aspects of diabetes(9 items), assessing dissatisfaction and readiness to change (9 items), and setting and achieving diabetic goals (10 items) [16]. The score of each item will range from 1 to 5 (1 = strongly disagree, 2 = disagree, 3 = neutral, 4 = agree, 5 = strongly agree), making a minimum score of 28 and the maximum score of 140. This range of score can be classified further into 3 groups: low empowerment group (28–65 score), medium empowerment group (66–103 score) and high empowerment group (104–140).

Diabetes Quality of Life (DQOL) Questionnaire is used to assess the quality of life for adults with diabetes [26]. This questionnaire consists of 46 questions in reflecting four sections. Section 1: This section is designed to test the patient’s satisfaction toward the life as a diabetic patient. This section consists of 15 questions. Section 2: This section consists of questions that will access the diabetes impacts on the patient’s social and daily life. This section consists of 20 questions. Section 3: This section consists of 11 questions that are divided into 2 parts. The first part includes the first 7 questions to access the patient’s worry towards social and vocational problems related to diabetes. Part 2 includes the remaining 4 questions to access the patient’s worry towards other diabetes related problems. It is a self-administered Likert type scale where 1 = very satisfied to 5 = very dissatisfied.

The Summary of Self-Care Diabetes Activities (SDSCA) questionnaire [19] is a self-reported scale that explores the levels of self-care among patients with diabetes over the past 7 days. The original questionnaire has 12 items which has been revised to 11 items later on. Patients were instructed to choose between 0 and 7 indicating days of diabetic specific behavior with higher scores indicating better performances of self-care activities. The score of each item will range from 1 to 5 (1 = strongly disagree, 2 = disagree, 3 = neutral, 4 = agree, 5 = strongly agree), making a minimum score of 28 and the maximum score of 140.

### Statistical analysis

The information from the questionnaire was collected and filled into master sheet using the Statistical Package for Social Science (SPSS) version 22. First, the suitability of the DES-M data for factor analysis was verified by using the Bartlett’s test of sphericity and the Kaiser-Mayer-Olkin measure of sampling adequacy. Second, parallel analysis was performed to obtain the suitable factors. Construct validity was investigated by exploratory factorial analysis (EFA) with varimax rotation. A factor loading of > 0.40 was used to determine the items for each factor. Third, PLS (partial least square) method using SMART-PLS 2 [27] was used for construct validity. Assessment of reflective measurement models includes some indicators such as composite reliability (CR), average variance extracted (AVE) and Cronbach’s alpha (α). Fourth, the Fornell-Larcker criterion [28] and cross loadings were used to assess discriminant validity. Fifth, all the unreliable observed indicator variables were removed from a measurement model that offers a poor fit to the data. Pearson correlation test was used to look for any relationship between these 2 questionnaires (DES and SDSCA), we performed a multiple linear regression analysis to examine predictive validity using the DQOL as dependent variable and the DES-M as independent variable.

## Results

The non-response rate was 25% and all of them did not give consent to join the study due to time constraint. In the end, a total of 151 diabetes patients were recruited into the analysis. Table 1 shows the demographic and medical background. Overall, the mean age was approximately 55 ± 13 years. More than half of the participants were male (66.2%) and two third were Malay ethnicity (61.6%). Majority of them married (92%) and received education at least secondary school and above (82.2%). More than half of the study participants have underlying hypertension (76.8%) and dyslipidaemia (60.3%). The mean duration of diabetes was 11 ± 9.43. On average, two third of the participants (66.2%) had not received any form of diabetes education program.

### Reliability and validity of DES-M

**Fig. 1:**
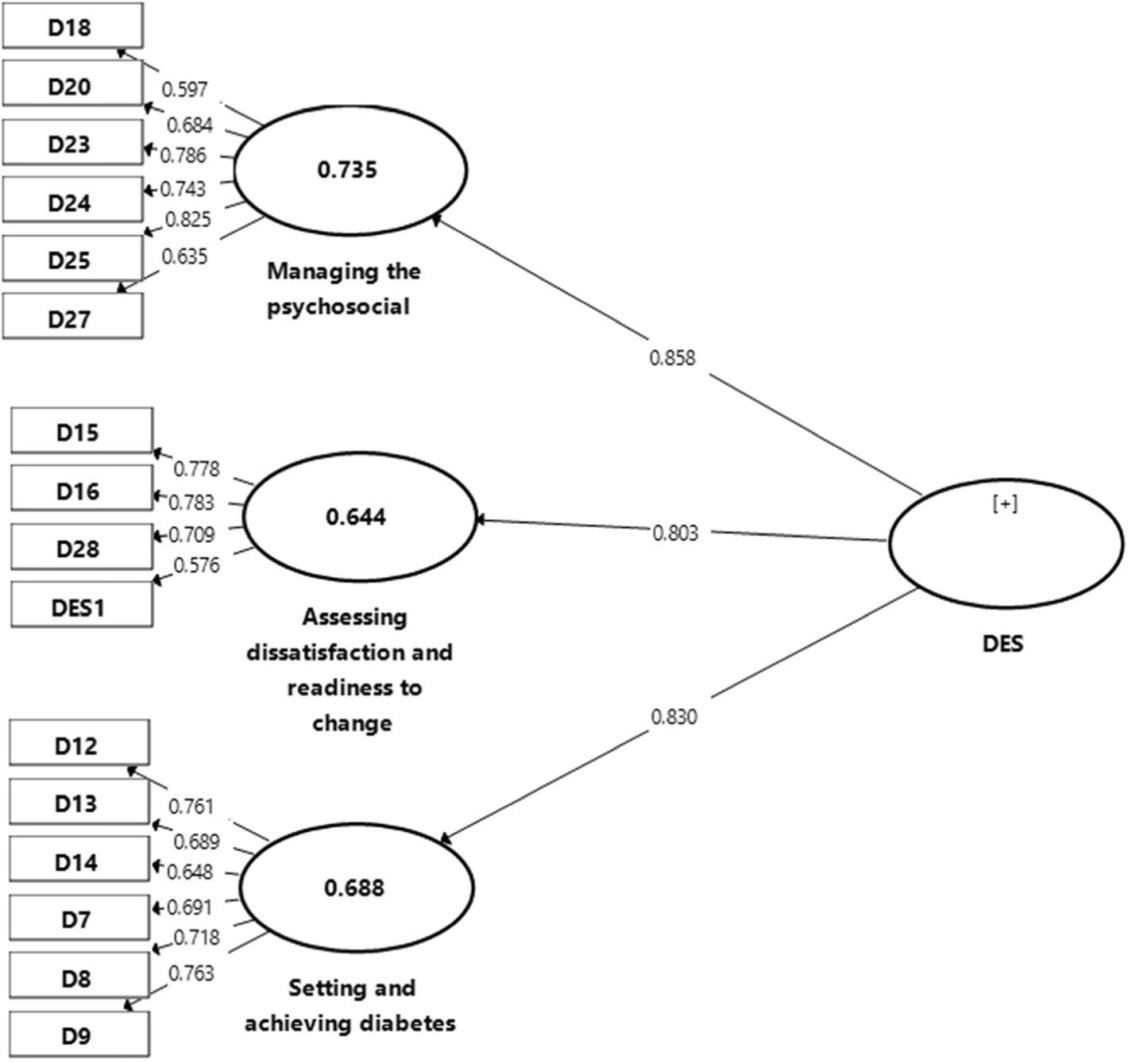
Confirmatory Factorial Analysis (CFA) in Three-factor model

**Fig. 2:**
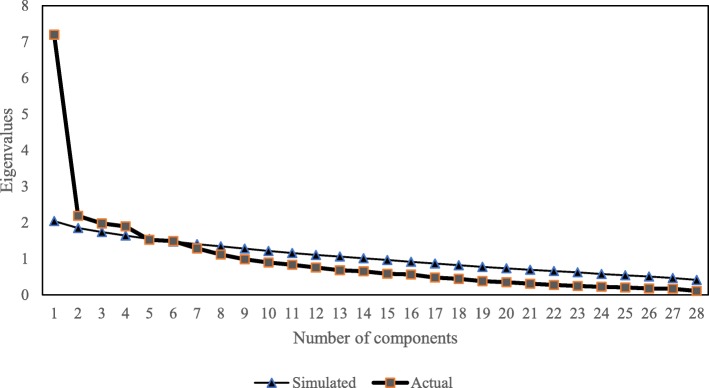
Parallel analysis

**Fig. 3:**
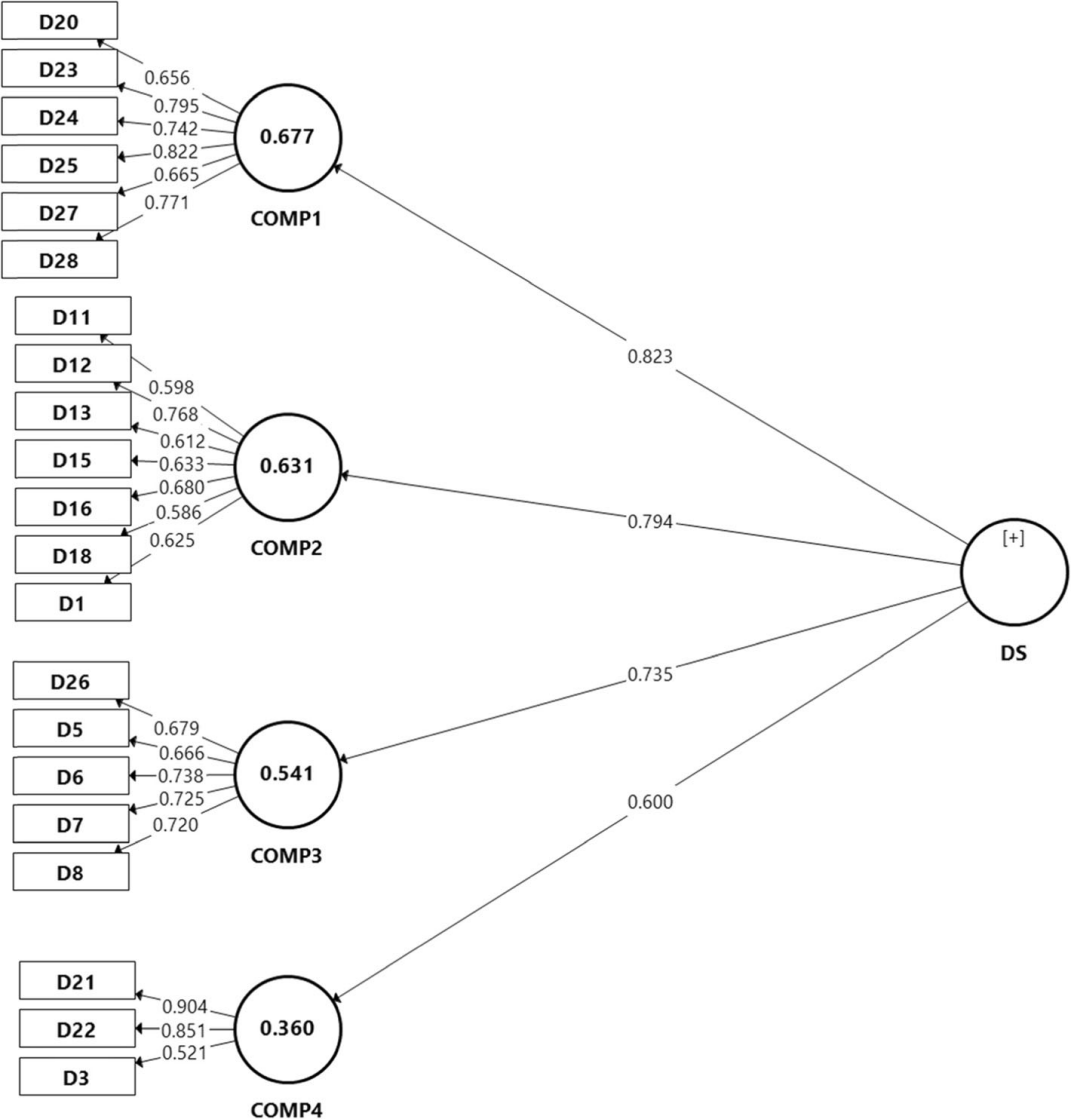
Exploratory Factorial Analysis of DES-M

While the confirmatory factorial analysis (CFA) were performed, 42% of the items were dropped due to low loading factors and convergent validity (Fig. 1) and Table 7 in Appendix. Therefore, EFA was conducted in order to find out the probable different pattern among items in the DES-M. The Bartlett’s test of sphericity was significant (*p* < 0.01) and the Kaiser-Mayer-Olkin measure of sampling adequacy for the DES-M was 0.76 indicating middling [29] and that factor analysis was appropriate. Using parallel analysis, this study obtained a four-factor model of the DES-M in 151 patients with diabetes mellitus (Fig. 2.) The four factors (Table 2.) which corresponded to the DES-M subscales referred to as “COMP 1”, “COMP 2”, “COMP 3”, “COMP 4”. Items with low loading and cross-loading were removed, resulting only 20 items left (Fig. 3.).

**Table 2:** Exploratory Factorial Analysis of DES-M

	Component			
Item	COMP1	COMP 2	COMP 3	COMP 4
D25	0.800			
D23	0.757			
D24	0.629			
D28	0.623			
D27	0.517			
D20	0.508			
D12		0.752		
D11		0.584		
D13		0.548		
D16		0.492		
D15		0.463		
D18		0.461		
DS1		0.400		
D19 ^a^		0.397		
D10 ^a^		0.393		
D17 ^a^		0.368		
D14 ^a^		0.329	0.319	
D6			0.764	
D5			0.610	
D26			0.541	
D7			0.540	
D9 ^b^		0.460	0.468	
D8			0.466	
D22				0.440
D21				0.430
D3				0.430
D2 ^a^				0.275
D4 ^a^				0.158

Extraction Method: Exploratory factor analysis

Rotation Method: Promax with Kaiser Normalization

^a^Deleted due to low loading factor

^b^deleted due to cross-loading

### Convergent validity and construct reliability

Table 3 showed the all items had an outer loading above 0.5 which were above the threshold. These results revealed that critical ratio (CR) was 0.81 to 0.88. In addition, in this study, Average Variance Extracted (AVE) for all the subscales was above 0.5. Cronbach’s alpha, which provides an estimate of the reliability based on the inter-correlations of the observed indicator variables also was more than threshold (0.5). Thus, the results proved that convergent validity and construct reliability existed for the constructs of this study.

**Table 3:** Results Summary for Measurement Model of DES-M (Convergent Validity)

	Cronbach’s Alpha	Composite Reliability	Average Variance Extracted (AVE)
COMP1	0.837	0.88	0.554
COMP2	0.765	0.833	0.417
COMP3	0.753	0.832	0.500
COMP4	0.649	0.813	0.604

*AVE* Average variance extracted, *CR* Composite reliability

The DES-M exhibited good internal consistency; Cronbach’s alpha coefficient for the total scale was 0.86, and the respective coefficients for the four factors were 0.83 for COMP1, 0.77 for COMP 2, 0.77 for COMP 3 and 0.60 for COMP 4.

### Discriminant validity

Based on Table 4, AVE for each construct is more than each of the squared correlation between constructs. Therefore, discriminant validity is adequate for all the constructs [28, 30]. the correlations between the latent variables ranged from 0.474 to 0.744, which were below the threshold 0.8, the squared correlations were less than the square root of the AVE by the indicators, hence, discriminant validity was established in this model [31].

**Table 4:** Correlation of latent variables and discriminant Validity of DES-M

	COMP1	COMP2	COMP3	COMP4
COMP1	**0.744**			
COMP2	0.586	**0.765**		
COMP3	0.527	0.567	**0.707**	
COMP4	0.497	0.524	0.474	**0.777**

Bold number = square root of the average variance extracted (AVE)

### Concurrent validity

The SDSDQ was used to evaluate the convergent validity of the DES in the present sample (Table 5). Correlation results indicated that there is a positive correlation between the DES-M and SDSDQ (*r* = 0.313, *p* = 0.012), thereby establishing concurrent validity.

**Table 5:** Pearson’s Correlation for Concurrent Validity of DES-M with SDSDQ

Correlations			
		Total DES	Total Score SDSDQ
Total DES	Pearson Correlation	1	.313^*^
	Sig. (2-tailed)		.012
Total Score SDSDQ	Pearson Correlation	.313^*^	1
	Sig. (2-tailed)		.012

### Predictive validity

In regard to predictive validity, the multiple regression analysis exhibited DES-M was statistically significant positive predictors for DQOL (B = 0.298, *p* < 0.001). DES-M total score accounted for 8.9% of the variance in patients’ quality of life score, F = 14.56, *p* < 0.05 (Table 6).

**Table 6:** Multiple Regression Analysis for Prediction of Diabetes Quality of Life

Variable	R	R^2^	F	d*f*	*B*	t	p	Tolerance	*VIF*
Total DQOL score	0.298	0.089	14.556	1	0.298	3.815	0.000	1.00	1.00

## Discussion

This study examined the internal consistency, concurrent and predictive validity of DES-M. The findings from this study indicate that the DES-M is a reliable and valid instrument for assessing diabetes empowerment in Malay-speaking population.

In this study, the DES-M exhibited a good internal consistency; Cronbach’s alpha coefficient for the total was 0.92, and the respective coefficients for the four sub-scales were 0.84, 0.75, 0.79 and 0.65. This is consistent with the findings with other studies such as the internal consistency of Sweden version of DES (Swe-DES-23) was 0.91 [32]; Iranian version of DES (IR-DES-28) was 0.89 [33] and Chinese version of DES (C-DES-20) was 0.86 [34]. To date, this is the first study of its kind related to Diabetes Empowerment scale in Malaysia and it shows that the DES-M is as good as Cronbach’s alpha coefficient as the English version and also others translated version of DES.

Criterion validity is used to determine how well of one measure in predicting the outcome for another measure. It can be classified into concurrent validity and predictive validity. Concurrent validity is defined as whether it measures what it is supposed to measure based on a well-established test. Concurrent validity was demonstrated by DES-M score positive correlated significantly to SDSCA (*r* = 0.313, *p* = 0.012), this result is coherent to Majouri et al., 2012, which IR-DES-28 was positively correlated to Diabetes attitude scale-33 (DAS-33)(*r* = 0.42, *p* < 0.01) [33].

Predictive validity is defined as the ability of an instrument in predicting the future health status of the participants and this appeared to be a better indicator for health-related outcome. Predictive validity was tested by using multiple regression linear regression, the result shows DES-M score could predict DQOL (B = 0.298, *p* < 0.001). Discriminant or divergent validity is defined as those items within any one subscale that highly distinctive with external items of another subscale [35]. Discriminant validity showed adequate correlations ranged from 0.257–0.744.

### Strength and limitations

To date, this is the first study on validating of DES-M in Malaysia which could assist future research to measure patient’s empowerment to improve the management and treatment towards diabetes. In addition, majority Malaysian are of Malay ethnic. Hence, this version of the questionnaire can help to fit in Malaysia’s setting and decrease misinterpretation of the questionnaire caused by language. There were a few limitations. First, this study was conducted in Hospital Serdang only without using randomisation. Thus, this study population cannot be generalized to all diabetic patients in Malaysia due to limitation on selection of sampling method. Besides that, the sample size of this study was conducted in slightly small sample size (*n* = 151) however acquiring more data does not necessary lead to more information and to data, there is no gold standard to get the sufficient sample size for the validation study as literature have included patients based on “subject to item” ratio a posteriori from 5 to 20 [35]. The convergent validity using SDSCA showed a weak to moderate correlation, therefore we need to interpret the result cautiously.

## Conclusions

This study developed the Diabetes empowerment scale for diabetes patient among Malaysian population. This study also provide evidence that the DES-M is a valid and reliable, self-administered tool to measure self-empowerment among patient with diabetes.

## Data Availability

The datasets generated and analyzed in Department of Family Medicine, Faculty of Medicine and Health Sciences, Universiti Putra Malaysia. This study is available in Department of Family Medicine, Faculty of Medicine and Health Sciences, Universiti Putra Malaysia.

## Appendix

**Table 7:** Confirmatory Factorial Analysis (CFA) in Three-factor model

Domain	Item	Outer loading		Cronbach’s Alpha	Composite Reliability	Average Variance Extracted (AVE)
		Initial model	Modified model			
Setting and achieving diabetes	D5	0.49	Deleted	0.805	0.861	0.508
	D6	0.512	Deleted			
	D10	0.524	Deleted			
	D11	0.529	Deleted			
	D13	0.59	0.689			
	D14	0.608	0.648			
	D7	0.678	0.691			
	D12	0.717	0.761			
	D8	0.723	0.718			
	D9	0.763	0.763			
Assessing dissatisfaction and readiness to change	D4	0.021	Deleted	0.681	0.806	0.513
	D2	0.093	Deleted			
	D3	0.312	Deleted			
	D17	0.42	Deleted			
	D19	0.523	Deleted			
	DES1	0.593	0.576			
	D28	0.617	0.709			
	D15	0.754	0.778			
	D16	0.754	0.783			
Managing the psychosocial	D26	0.532	Deleted	0.806	0.862	0.513
	D21	0.534	Deleted			
	D22	0.548	Deleted			
	D18	0.555	0.597			
	D20	0.646	0.684			
	D27	0.658	0.635			
	D24	0.711	0.743			
	D23	0.739	0.786			
	D25	0.758	0.825			

## Abbreviations

AVE: Average variance extracted
CFA: Confirmatory factor analysis
CR: Critical ratio
DES-M: Diabetes empowerment scale malay version
DQOL: Diabetes quality of life for adults
EFA: Exploratory factor analysis
SDSCA: Summary of diabetes self care activities questionnaire

## References

[1] Letchuman G, Wan Nazaimoon W, Wan Mohamad W, Chandran L, Tee G, Jamaiyah H, Isa M, Zanariah H, Fatanah I, Ahmad Faudzi Y (2010). Prevalence of diabetes in the Malaysian national health morbidity survey III 2006. Med J Malaysia.

[2] Zhang P, Zhang X, Brown J, Vistisen D, Sicree R, Shaw J, Nichols G (2010). Global healthcare expenditure on diabetes for 2010 and 2030. Diabetes Res Clin Pract.

[3] Mastura I, Chew BH, Lee PY, Cheong AT, Sazlina SG, Jamaiyah H, Syed Alwi S, Sri Wahyu T, Zaiton A (2011). Control and treatment profiles of 70,889 adult type 2 diabetes mellitus patients in Malaysia-a cross sectional survey in 2009. Int J Collaborative Res Intern Med Public Health.

[4] Vincent D, McEwen MM, Pasvogel A (2008). The validity and reliability of a Spanish version of the summary of diabetes self-care activities questionnaire. Nurs Res.

[5] Zhu TH, Mooi CS, Shamsuddin NH (2019). Diabetes empowerment scores among type 2 diabetes mellitus patients and its correlated factors: a cross-sectional study in a primary care setting in Malaysia. World J Diabetes.

[6] Goodall TA, Halford WK (1991). Self-management of diabetes mellitus: a critical review. Health Psychol.

[7] Johnson SB (1980). Psychosocial factors in juvenile diabetes: a review. J Behav Med.

[8] Bridges J.F.P., Loukanova S., Carrera P. (2008). Patient Empowerment in Health Care. International Encyclopedia of Public Health.

[9] Stuart K, Borland R, McMurray N (1994). Self-efficacy, health locus of control, and smoking cessation. Addict Behav.

[10] Dennis KE, Goldberg AP (1996). Weight control self-efficacy types and transitions affect weight-loss outcomes in obese women. Addict Behav.

[11] DuCharme KA, Brawley LR (1995). Predicting the intentions and behavior of exercise initiates using two forms of self-efficacy. J Behav Med.

[12] Funnell MM, Anderson RM, Arnold MS, Barr PA, Donnelly M, Johnson PD, Taylor-Moon D, White NH (1991). Empowerment: an idea whose time has come in diabetes education. Diabetes Educ.

[13] Littlefield CH, Craven JL, Rodin GM, Daneman D, Murray MA, Rydall AC (1992). Relationship of self-efficacy and binging to adherence to diabetes regimen among adolescents. Diabetes Care.

[14] Hurley AC, Shea CA (1992). Self-efficacy: strategy for enhancing diabetes self-care. Diabetes Educ.

[15] Tol A, Baghbanian A, Mohebbi B, Shojaeizadeh D, Azam K, Shahmirzadi SE, Asfia A (2013). Empowerment assessment and influential factors among patients with type 2 diabetes. J Diabetes Metab Disord.

[16] Anderson RM, Funnell MM, Fitzgerald JT, Marrero DG (2000). The diabetes empowerment scale: a measure of psychosocial self-efficacy. Diabetes Care.

[17] Schmitt A, Gahr A, Hermanns N, Kulzer B, Huber J, Haak T (2013). The diabetes self-management questionnaire (DSMQ): development and evaluation of an instrument to assess diabetes self-care activities associated with glycaemic control. Health Qual Life Outcomes.

[18] Toobert DJ, Glasgow RE (1994). Assessing diabetes self-management: the summary of diabetes self-care activities questionnaire. Handb Psychol Diabetes: Guid Psychol Meas Diabetes Res Pract.

[19] Toobert DJ, Hampson SE, Glasgow RE (2000). The summary of diabetes self-care activities measure: results from 7 studies and a revised scale. Diabetes Care.

[20] Kamradt M, Bozorgmehr K, Krisam J, Freund T, Kiel M, Qreini M, Flum E, Berger S, Besier W, Szecsenyi J (2014). Assessing self-management in patients with diabetes mellitus type 2 in Germany: validation of a German version of the summary of diabetes self-care activities measure (SDSCA-G). Health Qual Life Outcomes.

[21] AlJohani KA, Kendall GE, Snider PD (2016). Psychometric evaluation of the summary of diabetes self-care activities–Arabic (SDSCA-Arabic) translation and analysis process. J Transcult Nurs.

[22] Kav S, Akman A, Dogan N, Tarakci Z, Bulut Y, Hanoglu Z (2010). Turkish validity and reliability of the summary of diabetes self-care activities measure for patients with type 2 diabetes mellitus. J Clin Nurs.

[23] Choi EJ, Nam M, Kim SH, Park CG, Toobert DJ, Yoo JS, Chu SH (2011). Psychometric properties of a Korean version of the summary of diabetes self-care activities measure. Int J Nurs Stud.

[24] Bujang MA, Ismail M, Bariyyah N (2016). Validation of the summary of diabetes self-care activities (SDSCA) in Malay language for Malaysian adults. MJPHM.

[25] Organization WH: Process of translation and adaptation of instruments**.** 2007. In*.*; 2010.

[26] Group DR (1988). Reliability and validity of a diabetes quality-of-life measure for the diabetes control and complications trial (DCCT). Diabetes Care.

[27] Ringle CM, Wende S, Will A. Smart PLS 2.0 M3. Germany: University of Hamburg: Book Smart Pls. 2005;2:M3.

[28] Fornell Claes, Larcker David F. (1981). Structural Equation Models with Unobservable Variables and Measurement Error: Algebra and Statistics. Journal of Marketing Research.

[29] Kaiser HF (1974). An index of factorial simplicity. Psychometrika.

[30] Hair JF, Black WC, Babin BJ, Anderson RE, Tatham RL (2006). Multivariate data analysis (Vol. 6). In*.*: upper Saddle River.

[31] Kline RB. Principles and practice of structural equation modeling: Guilford publications; 2015.

[32] Leksell J, Funnell M, Sandberg G, Smide B, Wiklund G, Wikblad K (2007). Psychometric properties of the Swedish diabetes empowerment scale. Scand J Caring Sci.

[33] Mahjouri MY, Arzaghi SM, Heshmat R, Khashayar P, Esfahani EN, Larijani B (2012). Psychometric properties of the Iranian version of diabetes empowerment scale (IR-DES-28). J Diabetes Metab Disord.

[34] Shiu AT, Wong RY, Thompson DR (2003). Development of a reliable and valid Chinese version of the diabetes empowerment scale. Diabetes Care.

[35] Anthoine E, Moret L, Regnault A, Sébille V, Hardouin J-B (2014). Sample size used to validate a scale: a review of publications on newly-developed patient reported outcomes measures. Health Qual Life Outcomes.

